# Ubiquitin Degradation of the AICAR Transformylase/IMP Cyclohydrolase Ade16 Regulates the Sexual Reproduction of *Cryptococcus neoformans*

**DOI:** 10.3390/jof9070699

**Published:** 2023-06-24

**Authors:** Liantao Han, Yujuan Wu, Sichu Xiong, Tongbao Liu

**Affiliations:** 1 State Key Laboratory of Resource Insects, Southwest University Medical Research Institute, Chongqing 400715, China; hlt892996713@email.swu.edu.cn (L.H.); wyj0213@email.swu.edu.cn (Y.W.); xiongsc@email.swu.edu.cn (S.X.); 2 Jinfeng Laboratory, Chongqing 401329, China

**Keywords:** *Cryptococcus neoformans*, sexual reproduction, F-box protein Fbp1, AICAR transformylase/IMP cyclohydrolase, Ade16

## Abstract

F-box protein is a key protein of the SCF E3 ubiquitin ligase complex, responsible for substrate recognition and degradation through specific interactions. Previous studies have shown that F-box proteins play crucial roles in *Cryptococcus* sexual reproduction. However, the molecular mechanism by which F-box proteins regulate sexual reproduction in *C. neoformans* is unclear. In the study, we discovered the AICAR transformylase/IMP cyclohydrolase Ade16 as a substrate of Fbp1. Through protein interaction and stability experiments, we demonstrated that Ade16 is a substrate for Fbp1. To examine the role of *ADE16* in *C. neoformans*, we constructed the i*ADE16* strains and *ADE16*^OE^ strains to analyze the function of Ade16. Our results revealed that the i*ADE16* strains had a smaller capsule and showed growth defects under NaCl, while the *ADE16*^OE^ strains were sensitive to SDS but not to Congo red, which is consistent with the stress phenotype of the *fbp1*Δ strains, indicating that the intracellular protein expression level after *ADE16* overexpression was similar to that after *FBP1* deletion. Interestingly, although i*ADE16* strains can produce basidiospores normally, *ADE16*^OE^ strains can produce mating mycelia but not basidiospores after mating, which is consistent with the *fbp1*Δmutant strains, suggesting that Fbp1 is likely to regulate the sexual reproduction of *C. neoformans* through the modulation of Ade16. A fungal nuclei development assay showed that the nuclei of the *ADE16*^OE^ strains failed to fuse in the bilateral mating, indicating that Ade16 plays a crucial role in the regulation of meiosis during mating. In summary, our findings have revealed a new determinant factor involved in fungal development related to the post-translational regulation of AICAR transformylase/IMP cyclohydrolase.

## 1. Introduction

*Cryptococcus neoformans* is a yeast-like pathogenic fungus that is widely found in natural environments such as soil, plant surfaces, and bird feces [1]. The basidiospores or dried yeast cells of *C. neoformans* can be inhaled into the host’s lungs through the respiratory tract where they are eliminated or stay in a state of latent infection in immunocompetent hosts [2]. When the host’s immunity is impaired, or the hosts are immunocompromised individuals, *C. neoformans* can proliferate in the lungs of the host and cause cryptococcal pneumonia, penetrating the blood–brain barrier (BBB) to cause deadly cryptococcal meningitis [3]. Statistically,19% of the deaths related to acquired immune deficiency syndrome (AIDS) are caused by cryptococcal meningitis, and about 112,000 cases of cryptococcal meningitis are caused yearly [4].

As a basidiomycete, *C. neoformans* has two mating types, α and **a**, which can reproduce sexually or asexually [5]. Typically, *C. neoformans* grows as budding yeast and can switch from yeast growth to filamentous growth by mating and monokaryotic fruiting [6,7]. The transition of morphotypes from yeast to hyphae marks the beginning of the sexual development of *C. neoformans* [8]. During the mating of *C. neoformans*, the yeast cells of different mating types fuse and form dikaryotic mating hyphae, and eventually, a basidium, the specialized sporulation structure, is produced at the tip of the hyphae. Then, after completing meiosis inside the basidium, four chains of basidiospores are generated at the top of the basidium [7,8]. Similar environmental factors also affect the monokaryotic fruiting of *C. neoformans* but occur between the haploid yeast cells of the same mating type, such as α cells [9,10]. Compared with mating, the hyphal cells produced by monokaryotic fruiting are mononucleate diploid cells with unfused clamp connections [9].

Sexual reproduction of *C. neoformans* is regulated by many environmental factors and genetic circuits. Light, darkness, ambient temperature, nutrition deficiency, pheromone, and metal ions regulate cell–cell fusion and mating hypha growth during the sexual reproduction of *C. neoformans* [6,10], and the mechanisms of these environmental factors affecting the sexual cycle are usually rooted in gene regulatory circuits. The genetic circuits, such as the Cpk1 MAPK pathway and the pheromone response pathway, also regulate the sexual reproduction of *C. neoformans*, as the mutation of components in these pathways blocks the sexual reproduction of *C. neoformans* [6,11,12,13,14]. Besides these pheromone response pathways, other factors, such as zinc finger proteins, have also been identified as common regulators for sexual reproduction in *C. neoformans* [13,15,16].

The ubiquitin-proteasome system (UPS) is the main protein degradation system in eukaryotic cells, responsible for the degeneration of more than 80% of protein and maintaining intracellular protein homeostasis [17,18]. The Cullin1, Skp1, and F-box protein (SCF) E3 ligases are the most prominent E3 ubiquitin ligases, in which the F-box protein specifically recognizes downstream substrate proteins and meditates ubiquitination degradation of various substrate proteins [18]. Our previous studies showed that SCF E3 ligases play a vital role in the sexual reproduction of *C. neoformans*, as disruption of the key proteins of SCF E3 ligases such as Fbp1 and Cdc4 block basidiospore production in *C. neoformans* [19,20]. However, the molecular mechanism by which the F-box protein regulates *C. neoformans* sexual reproduction remained unclear. AICAR transformylase/IMP cyclohydrolase (ATIC) is a bifunctional, rate limiting enzyme that catalyzes the last two steps of the de novo purine biosynthesis pathway and plays an important role in cell proliferation and metabolism [21]. *C. neoformans* mutants lacking the ATIC-encoding *ADE16* gene are adenine and histidine auxotrophic and are unable to infect mice [22]. Here, we show that the ATIC Ade16 *of C. neoformans* is a downstream target of Fbp1. Overexpression of the *ADE16* gene blocks basidiospore formation by affecting the nuclear fusion of the meiosis process during mating. Our findings uncover a new determinant of fungal development involving the post-translational regulation of an AICAR transformylase/IMP cyclohydrolase.

## 2. Materials and Methods

### 2.1. Strains and Growth Conditions

Wild-type strains of *C. neoformans*, H99, and KN99**a**, and their derived strains were preserved routinely in our laboratory and cultured on a YPD agar or liquid medium at 30 °C. *C. neoformans* strains expressing the genes controlled by the *CTR4* promoter were cultured on a YPD medium supplemented with 200 μM bathocuproinedisulfonic acid (BCS) or 25 μM CuSO_4_ with 1 mM ascorbic acid [23]. The yeast strain used in the yeast two-hybrid assay was Y187, whose transformants were cultured on a synthetic defined medium without leucine, tryptophan, histidine, or adenine. The *C. neoformans* and yeast strains used in the study are shown in Table S1. All other media used in the study were prepared as described previously [19].

### 2.2. Quantitative Real-Time PCR

To detect the *ADE16* gene expression under different conditions, quantitative real-time PCR (qRT-PCR) was used to measure the mRNA expression levels of *ADE16* as previously described [24]. Briefly, yeast cells or mating mixtures of each cryptococcal strain were collected and washed with distilled water (dH_2_O); total RNA was extracted and purified using an Omega total RNA kit (Omega Bio-tek, Norcross, GA, USA). The purified RNAs were quantified using a Nanodrop spectrometer (DeNovix, Wilmington, DE, USA), and first-strand cDNA synthesis was synthesized with a Hifair^®^ II 1st Strand cDNA Synthesis Kit (Yeasen, Shanghai, China), as described by the manufacturer. The analysis of *ADE16* gene expression was performed using FastStart Essential DNA Green Master (Roche, Mannhein, Germany), and the gene expression levels were normalized using the endogenous control gene *GAPDH*. Relative gene expression levels were calculated using the previously described comparative threshold cycle (CT) method [25]. The qRT-PCRs were carried out using a LightCycler^®^96 QPCR system (Roche) as described previously [26].

### 2.3. Generation of Fluorescence Strains

To detect the *ADE16* gene expression in *C. neoformans* at different developmental stages, we amplified a 1996-bp *ADE16* gene promoter sequence using H99 genomic DNA as a template with primers TL1166/1167 (see Table S2 for primer sequences) and inserted it into the pTBL5 [16] plasmid to construct the plasmid pTBL196. Then, the pTBL196 plasmid was linearized by *Sal*I, concentrated, and biolistically introduced into the wild-type strains, H99 and KN99**a**, respectively, as previously described [27]. Stable transformants were further screened by growing on YPD plates with nourseothricin sulfate (100 mg/L) and fluorescence examination under an Olympus inverted confocal microscope (Olympus, FV1200). Finally, two mating types of the P*_ADE16_*-mCherry fluorescence fusion expression strains (α and **a**) were obtained and named TBL310 and TBL378, respectively.

To determine Ade16 sub-cellular localization, we amplified the *ADE16* gene coding sequence from the H99 genomic DNA using primers TL1164/TL1165 and inserted it into the pCN19 vector to construct the GFP and *ADE16* fusion gene expression vector pTBL186. The *Xba*I-linearized pTBL186 was biolistically introduced into the H99 or KN99**a** strains, respectively. Stable transformants were further confirmed on YPD plates with nourseothricin sulfate (100 mg/L) and named TBL308 and TBL309, respectively. The fluorescence of the transformants was examined via confocal microscopy using a 100× objective lens (Olympus, FV1200, Tokyo, Japan). 

### 2.4. Yeast Two-Hybrid Assays

We first carried out a yeast two-hybrid interaction assay to detect the interaction between Ade16 and Fbp1 proteins as described previously [19,28]. The full-length cDNAs of the *ADE16* and *FBP1* genes were amplified with primers TL879/880 and 588/589 and cloned into the bait vector pGBKT7 to fuse with the BD domain (pTBL106 and pTBL100), respectively. Meanwhile, the cDNAs of *ADE16* and *FBP1* were also amplified with primers TL883/884 and TL572/573 and inserted into the pGADT7 prey vector (pTBL145 and pTBL142), respectively, fusing with the AD domain. The inserted cDNAs were verified by sequencing to ensure the proteins were translated correctly. Both prey and bait constructs were co-transformed into the yeast strain Y187. After transformation, the yeast cells were transferred to an SD-Leu-Trp plate and incubated for 2–3 days at 30 °C. The transformants growing on SD-Leu-Trp-His or SD-Leu-Trp-His-Ade plates were considered positive interactions. The transformants carrying pGADT7-T7/pGBKT7-LAM and pGADT7-T7/pGBKT7-53 served as negative and positive controls, respectively (Clontech, Dalian, China).

### 2.5. Generation of Tagged Protein Strains

To obtain *C. neoformans* strains expressing the Ade16-HA protein, we first amplified the *ADE16* cDNA with primers TL893/894. We then cloned it into the *Bam*HI site of the vector pCTR4-2 [23] to generate the Ade16:HA fusion plasmid pTBL149 using the In-Fusion HD cloning kit (Clontech, Dalian, China). The *C. neoformans* strain (TBL81) expressing the Fbp1-Flag fusion protein was generated by our previous studies [29]. To detect the interaction between Ade16 and Fbp1 in *C. neoformans*, we biolistically introduced the *EcoR*I-linearized pTBL149 into the Fbp1-Flag strain TBL81 and the wild-type H99, generating the *C. neoformans* strains TBL248 and TBL264, respectively, which express Fbp1:Flag/Ade16:HA protein and Ade16:HA protein.

Total proteins were extracted and examined using Western blotting with anti-HA or anti-Flag antibodies to ensure the correct tagged strains were obtained. Protein pull-down was performed using SureBeads™ anti-HA or anti-Flag Magnetic Beads (Bio-RAD) as described previously [24] to collect the tagged proteins, and immunoblotting was used to detect the Ade16-HA or Fbp1-Flag proteins with anti-HA or anti-Flag antibodies, respectively.

To detect the Ade16 stability in the H99 and *fbp1*Δ mutant strains’ backgrounds, we introduced the *EcoR*I-linearized pTBL149 into the *fbp1*Δ mutant biolistically to construct the Ade16:HA strain TBL264 and TBL265. Then, the yeast cells of TBL264 and TBL265 strain were grown in liquid YPD to mid-logarithmic phase, transferred to YPD containing 25 μM CuSO_4_ and 1 mM ascorbic acid, and incubated for an additional 1, 2, 4, and 6 h. Yeast cells were harvested, and protein extracts were prepared as described above. The signals of Ade16:HA was detected via Western blotting using a monoclonal anti-HA antibody (Sigma, Saint Louis, MO, USA).

### 2.6. Generation of ADE16 Interference and Overexpression Strains

To generate the *ADE16* promoter replacement strain, we used a split marker strategy to replace the *ADE16* promoter with a copper-repressible *CTR4* promoter. In the first round of PCR, as shown in Figure 3A, the 5′ fragment, the *NEO* marker, and the P_CTR4_-*ADE16* fusion fragment were amplified with primers TL1036/TL1037, TL17/TL18, and TL1034/TL1038 using the H99 genomic DNA, pJAF1, and pTBL149 as templates, respectively. In the next round of PCR, the fusion fragment of the 5′ fragment and *NEO* marker was amplified with primers TL1036/TL20 using the mixture of the 5′ fragment and *NEO* marker as templates. Similarly, using the mixture of the *NEO* marker and P*_CTR4_-ADE16* fragment as templates, the fusion fragment of the *NEO* marker and the P_CTR4_-*ADE16* fragment were amplified with primers TL19/TL1038. The above two fusion fragments were mixed, concentrated, and biolistically introduced into the H99 strain to construct the P*_CTR4_-ADE16* strain TBL270. The TBL270 strain was further confirmed via PCR with primers TL373/TL59 and Southern blotting. To verify whether *ADE16* is an essential gene for *C. neoformans*, we inoculated the H99 and TBL270 strain onto YPD, YPD + 200 μM BCS, or YPD + 25 μM CuSO_4_ + 1 mM ascorbic acid plates, respectively, after serial dilution. The growth of each cryptococcal strain was observed after 2–3 days of incubation at 30 °C.

To generate the *ADE16* RNAi-interference strains, we first constructed plasmid P_i*ADE16*_ for RNA interference of the *ADE16* gene in three steps. Step I: a 200-bp intron of the *ADE16* gene was amplified using primers TL1334/1335 and inserted into the *Bam*HI/*Pac*I sites of pTBL5 to generate the plasmid pTBL226. Step II: a 480-bp *ADE16* 5′–3′ fragment of the *ADE16* RNA interference sequence was amplified using primers TL1336/1337 and inserted into the *Bam*HI site of pTBL226 to generate pTBL227. Step III: a reversed 480-bp *ADE16* RNA interference sequence was amplified using primers TL1467/1468 and inserted into the *Pac*I/*Spe*I sites of pTBL227 to generate pTBL237. After verification by sequencing, *Sal*I-linearized pTBL237 was biolistically introduced into the wild-type strains H99 and KN99**a**. After PCR verification with primers TL1336/1340, the expression level of the *ADE16* gene was quantified using quantitative real-time PCR in each transformant, and two mating types of *ADE16* interference strains were named TBL414 and TBL415, respectively.

To obtain the *ADE16* gene overexpression strain in *C. neoformans*, we first amplified the *ADE16* gene using primers TL1092/1093 and inserted it into the *Bam*HI site of the pTBL153 plasmid to generate pTBL174. Then, the *Apa*I-linearized pTBL174 was biolistically introduced into the wild-type H99 and KN99**a** strains. Stable transformants were further confirmed on a YPD medium containing 100 mg/L nourseothricin sulfate. The *ADE16* gene overexpression (TBL288 and TBL302) was verified via qRT-PCR. To monitor the nuclear positioning in the *ADE16* overexpression strains during mating, pTBL59, containing a *NOP1-mCherry-NEO* cassette, was biolistically transformed into the *ADE16* overexpression strain of both α and **a** mating types to generate TBL445 and TBL446.

### 2.7. Analysis of Melanin and Capsule

To examine the role of Ade16 in *C. neoformans* melanin production, we grew the wild-type H99 strain and the Ade16-related strains in a YPD liquid medium overnight at 30 °C. One hundred microliters of each culture was inoculated on Niger seed medium to evaluate the melanin production; Niger seed agar plates were incubated for 2–3 days at 30 °C or 37 °C. The pigmentation of each cryptococcal strain was evaluated and photographed. 

To assess the role of Ade16 in *C. neoformans* capsule production, overnight cultures of each cryptococcal strain were washed with 1× PBS buffer three times and incubated overnight in diluted Sabouraud medium (DSM) or MM with mannitol at 37 °C [30,31]. The quantification of the capsule size was performed as previously described [19].

### 2.8. Mating Assay

To investigate the role of Ade16 in *C. neoformans* mating, equal amounts of the α and **a** mating type yeast cells were mixed and grown on MS or V8 agar plates in the dark at 25 °C. After 14 days of incubation, the formation of mating hyphae and basidiospores was visualized and recorded using an Olympus CX41 light microscope. 

### 2.9. Statistical Analysis

All statistical analyses were performed using Prism8.0 (GraphPad Software Inc., San Diego, CA, USA). Experiments consisting of only two groups were assessed with the Mann–Whitney test. Experiments involving a time series were compared using a Wilcoxon signed-rank test. Experiments involving multiple groups were analyzed with the nonparametric Kruskal–Wallis test. All data are represented with mean ± SD, unless specified in the figure legend. *p*-values of < 0.05 were considered statistically significant.

## 3. Results 

### 3.1. Interaction Profile and Expression Pattern of ADE16 during Mating of C. neoformans

Our previous study revealed that the F-box protein Fbp1, a key protein of the E3 ligase family, plays a crucial role in *C. neoformans* sexual reproduction, as in the bilateral mating of *fbp1*Δ mutants; basidiospore production was blocked despite the observation of normal dikaryotic hyphae during mating [19]. To identify downstream targets of Fbp1 in *C. neoformans*, we conducted an iTRAQ analysis coupled with LC-MS/MS in a previous study searching for enriched proteins in *fbp1*Δ mutants [24]. The partially enriched proteins are shown in Table 1 and have been selected as candidates for further study. 

**Figure 1:**
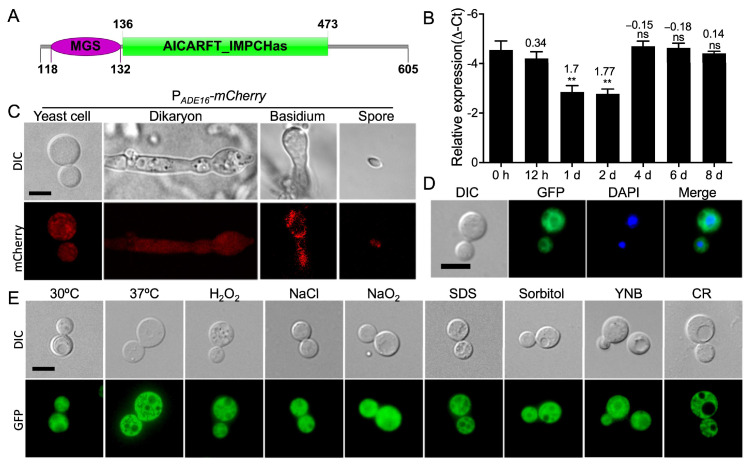
Identification of *C. neoformans* Ade16. Schematic illustration of the Ade16 protein (**A**) in *C. neoformans*. MGS: methylglyoxal synthase domain; AICARFT_IMPCHas: AICARFT/IMPCHase bienzyme domain. (**B**) The *ADE16* expression during bilateral mating on the V8 medium was qualified using qRT-PCR. The error bars represent the standard deviations of three independent repeats. ns, not significant; ** *p* < 0.01 (determined using Wilcoxon signed-rank test). Expression of mCherry (**C**) under the control of *ADE16* promoter in *C. neoformans* different development stages. Representative fluorescence and bright-field images of the yeast cells, dikaryons, basidia, and basidiospores are shown. Bar, 5 µm. (**D**) Subcellular localization of the GFP-Ade16 fusion protein in yeast cells of *C. neoformans*. DAPI staining was performed as previously described [19]. Bar, 5 µm. (**E**) Subcellular localization of the GFP-Ade16 fusion protein in yeast cells of *C. neoformans* under different stressors. DIC, differential interference contrast; Bar, 5 µm.

One of the candidate genes, CNAG_00700, was highly enriched in *fbp1*Δ mutants. Recently, Wizrah et al. identified the CNAG_00700 gene in *C. neoformans* and named it *ADE16* [22]. Sequence analysis revealed that the Ade16 protein contains two domains; one is the MGS (methylglyoxal synthase) domain, and the other is the AICARFT_IMPCHas (AICARFT/IMPCHase bienzyme) domain (Figure 1A). To investigate the function of *C. neoformans* Ade16 during fungal development, we detected *ADE16* gene expression H99 and KN99**a** strains using qRT-PCR. Expression of *ADE16* was significantly upregulated during the mating process and reached its highest value at the 2 d time point, then returning to comparable expression to the 0 h timepoint, suggesting that Ade16 plays a role in the sexual reproduction of *C. neoformans* (Figure 1B).

To more precisely observe the *ADE16* expression in *C. neoformans* at different developmental stages, we generated fluorescence strains (TBL310 and TBL378, see Table S2) expressing mCherry under the control of the native promoter of the *ADE16* gene in both mating types. The mCherry strains (TBL310 and TBL378) were crossed on MS medium and the fluorescence at different developmental stages of mCherry was visualized using a confocal microscope (Olympus, FV1200). mCherry was expressed in yeast cells, dikaryons, basidia, and basidiospores (Figure 1C), suggesting that Ade16 may play a key role in the fungal development of *C. neoformans*.

To detect Ade16 localization in *C. neoformans*, the *Xba*I-linearized *GFP-ADE16* plasmid was biolistically introduced into the wild-type H99 and KN99**a** strains to obtain TBL308 and TBL309. The expression of the GFP-Ade16 fusion protein was confirmed via Western blotting (Figure S1). The GFP-Ade16 fusion protein is localized in the cytoplasm of *C. neoformans* yeast cells and dikaryotic hyphae, except for the vacuoles (Figure 1D). Meanwhile, the subcellular localization of GFP-Ade16 in yeast cells was also examined under different stress conditions, and the results showed no difference among the GFP-Ade16 localizations under the above-tested stress conditions (Figure 1E).

### 3.2. Ade16 Interacts with Fbp1

**Figure 2:**
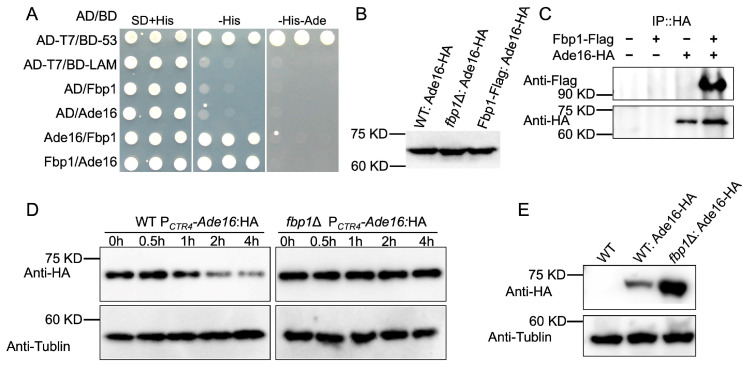
Ade16 interacts with Fbp1 and is a downstream target of Fbp1. (**A**) Ade16 interacts with Fbp1 in a yeast two-hybrid assay. The *ADE16* and *FBP1* full-length cDNAs were fused with the activation domain (AD) of the pGADT7 vector or the binding domain (BD) of the pGBKT7 vector. The two fusion vectors were transformed into the Y187 yeast host strain, and yeast colonies that grew on SD-Leu-Trp medium were further tested on SD-Leu-Trp-His and SD-Leu-Trp-His-Ade media. Yeast cells expressing AD-T7 and BD-53 fusion proteins were used as the positive controls, while that expressing AD-T7 and BD-LAM were used as the negative controls. (**B**) The expression of the Ade16-HA fusion protein in the wild-type H99, *fbp1*Δ mutants, or the Fbp1-Flag strains (TBL264, TBL265, and TBL248) was confirmed via Western blotting with anti-HA antibodies. (**C**) Ade16 interacts with Fbp1 in a Co-IP assay. Proteins from *C. neoformans* strains expressing Fbp1-Flag (TBL81), Ade16-HA (TBL270), or both Ade16-HA and Fbp1-Flag (TBL248) were extracted and purified. The potential interaction between Ade16 and Fbp1 was analyzed with Co-IP using anti-Flag (top panel) or anti-HA antibodies (bottom panel) and examined via Western blotting. (**D**) The Ade16 stability depends on the Fbp1 function. The *P_CTR4_-ADE16:HA* construct (pTBL149) was expressed in H99 (TBL264) or *fbp1*Δ mutants (TBL265). Yeast cells of TBL264 and TBL265 growing to the mid-logarithmic phase in YPD were collected at the indicated times after CuSO4 addition to stopping *ADE16* transcription, and the Ade16 abundance was detected with Western blotting with HA antibody. Tubulin was used as a loading control. (**E**) Detection of the abundance of Ade16 protein. Proteins from the yeast cells of the wild-type H99, the wild-type H99 expressing, or the *fbp1*Δ mutants expressing Ade16-HA were extracted and examined via Western blotting using an anti-HA antibody. The expression of the tubulin gene was used as a loading control.

Our previous proteomics data suggests that Ade16 is enriched in *fbp1*Δ mutants and might be a target for Fbp1-mediated ubiquitination (Table 1) [24]. We conducted a yeast two-hybrid protein–protein interaction assay to explore whether there is an interaction between Ade16 and Fbp1. Expression vectors containing Ade16 and Fbp1 fused to either the binding domain (BD) or the activation domain (AD) were co-transformed into the Y187 yeast strain, and transformants growing on SD-Leu-Trp-His or SD-Leu-Trp-His-Ade media were considered positive interactions. As shown in Figure 2A, Ade16 interacts with Fbp1 in a yeast two-hybrid protein–protein interaction system.

To further investigate the Ade16-Fbp1 interaction, we generated an Ade16:HA fusion expression vector (pTBL149) by cloning the *ADE16* gene into the pCTR4-2 plasmid, in which the expression of *ADE16* was regulated by the copper-repressible CTR4 promoter [23]. The resulting construct, pTBL149, linearized by *Eco*RI, was biolistically transformed into H99, the *fbp1*Δ mutants, and a previously constructed Fbp1:Flag overexpression strain [29]. The expression of Ade16:HA fusion proteins in each strain was confirmed via Western blot (Figure 2B). Total proteins from strains expressing Ade17:HA and Fbp1:Flag were purified with anti-HA Magnetic Beads (Bio-RAD), and Western blotted with anti-HA and anti-Flag antibodies. Both HA and Flag signals were detected from the co-immunoprecipitation (Co-IP) products, indicating that Ade16 interacts with Fbp1 in *C. neoformans* (Figure 2C).

### 3.3. The Ade16 Stability Depends on Fbp1 Function

To test our hypothesis that Ade16 is an Fbp1 downstream target, we investigated whether the Ade16 protein stability depends on the function of the SCF(Fbp1) E3 ligase. The Ade16:HA protein was expressed in the wild-type H99 strain (TBL264) and the *fbp1*Δ mutants (TBL265), and Ade16 protein stability was examined in the backgrounds of the above two strains. The Ade16:HA expressing strains were first cultured in a YPD medium containing 200 μM BCS to induce the *CTR4* promoter. Then, the cultures were washed with PBS and transferred to a YPD medium containing 1 mM ascorbic acid and 25 μM CuSO4 to block the transcription of *ADE16*:*HA*. The yeast cells were harvested after incubation for another 0, 1, 2, 4, and 6 h, and the abundance of the Ade16:HA protein was evaluated via Western blot. Our results of the protein stability assay revealed that the Ade16:HA protein was hydrolyzed in the wild-type H99 background in a time-dependent manner during the examined period (0 to 6 h) but was relatively stable in the *fbp1*Δ mutants, suggesting that the Ade16:HA stability is dependent on the function of Fbp1 (Figure 2D).

Next, to detect the abundance of Ade16-HA protein in *C. neoformans*, we extracted the Ade16-HA proteins from the H99 or *fbp1*Δ mutants and examined them using Western blotting using the anti-HA antibody. As shown in Figure 2E, the relative amount of Ade16-HA in *fbp1*Δ mutants increased compared to that of the H99 strain, suggesting that the degradation of Ade16 depends on the Fbp1 function in *C. neoformans*.

### 3.4. ADE16 Is Required for Normal Growth of C. neoformans

**Figure 3:**
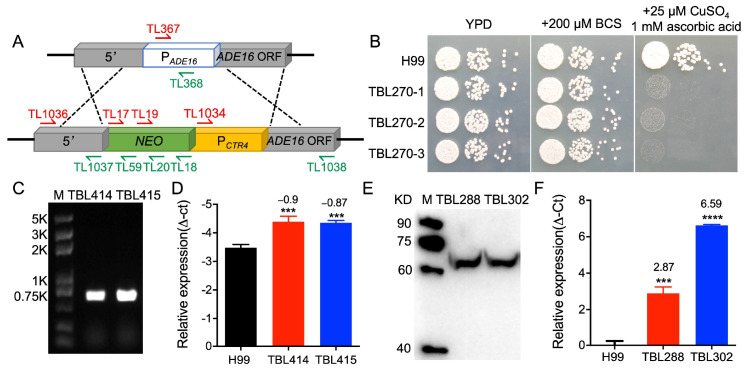
Generation of the *ADE16* interference strains and overexpression strains in *C. neoformans*. (**A**) The strategy of the *ADE16* native promoter substitution. 5′, ~1 kb DNA fragment upstream of *ADE16* gene promoter; P_ADE16_, *ADE16* native promoter; *ADE16* ORF, *ADE16* open reading frame; *NEO*, *NEO* marker gene; P_CTR4_, the copper-ion-suppressing CTR4 promoter. (**B**) The growth of the P*_CTR4_*-*ADE16* strains on YPD with 200 μM BCS and YPD with 25 μM CuSO_4_ and 1 mM ascorbic acid. Yeast cells grown overnight of the P*_CTR4_*-*ADE16* strains were diluted to an OD_600_ value of 2.0, followed by ten-fold dilutions. 5-µL of each diluent was plated on YPD agar plates with different stressors and incubated at 30 °C for 2 days. The *C. neoformans* strains are indicated on the left, and the incubation conditions are on the top. (**C**) PCR verification of the i*ADE16* interference strains (TBL414 and TBL415) with primers TL1336/1340. (**D**) Determination of the interference efficiency of the *ADE16* gene using qRT-PCR. The *ADE16* gene expression levels in the H99 strain grown on YPD were measured, and the *GAPDH* gene expression was used as an internal control. The relative expression of the *ADE16* was analyzed using ΔC(t) method. The data shown are the mean ± standard deviation from three repeats. ***, *p* < 0.001 (determined using the nonparametric Kruskal–Wallis test). (**E**) The expression of the Ade16-HA fusion protein in *ADE16*^OE^ overexpression strains (TBL288 and TBL302) was confirmed via Western blotting. (**F**) Overexpression of *ADE16*-*HA* TBL288 and TBL302 was also measured via relative qRT-PCR analysis. ***, *p* < 0.001; ****, *p* < 0.0001 (determined using the nonparametric Kruskal–Wallis test).

Wizrah et al. revealed that the *ADE16* null mutants are adenine and histidine auxotrophs and show severe growth defects on YPD [22]. To further confirm this phenotype, we decided to substitute the native promoter of the *ADE16* gene with the copper-ion-suppressing *CTR4* promoter to construct a *P_CTR4_-ADE16* strain (TBL270) and test its growth on YPD. The strategy of the *P_CTR4_-ADE16* strain construction is shown in Figure 3A. 

Overnight cultures of the wild-type H99 strain and *P_CTR4_-ADE16* strain (TBL270) were inoculated on YPD, YPD with 200 μM BCS, and YPD with 25 μM CuSO_4_ and 1 mM ascorbic acid and incubated at 30 °C for 2–3 days after series dilution. The growth of the *P_CTR4_-ADE16* strain was comparable to that of the wild-type strain when grown on YPD or YPD with 200 μM BCS. However, when grown on YPD with 25 μM CuSO_4_ and 1 mM ascorbic acid, the *P_CTR4_-ADE16* strain shows severe growth defects, while the growth capacity of the wild-type strain was not affected (Figure 3B), indicating that *ADE16* is required for the growth of *C. neoformans*.

### 3.5. ADE16 Overexpression Increases Capsule Size in C. neoformans

We constructed an *ADE16* RNA interference vector pTBL237 and introduced it into the *C. neoformans* wild-type strains to obtain the i*ADE16* interference strains (TBL414 and TBL415) in both α and **a** mating type. The construction of the *ADE16* interference strains and the *ADE16* gene interference efficiency was verified with PCR and qRT-PCR, respectively (Figure 3C,D). Meanwhile, the *ADE16*^OE^ overexpression strains (TBL288 and TBL302) were also constructed by introducing the *ADE16*^OE^ overexpression vector pTBL237 into the H99 and KN99**a** strains. The overexpression of *ADE16* in both mating types was confirmed via Western blot and qRT-PCR (Figure 3E,F).

**Figure 4:**
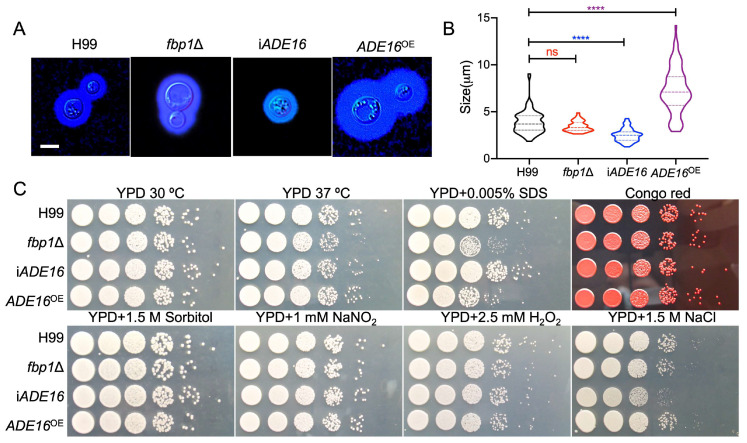
Ade16 is involved in the formation of *Cryptococcus* capsules. (**A**) Capsule formation of Ade16-related strains. Capsule formation was examined in a diluted SAB medium. Each *C. neoformans* strain was grown in a diluted SAB medium for 24 h at 37 °C, and the capsule formation was examined using India ink staining. Bars = 5 μm. (**B**) Statistical analysis of the capsule formation in each *C. neoformans* strain in diluted SAB medium. The capsule sizes of more than 100 yeast cells were measured, and the data shown are medians, quartile lines and ranges of the three replicates. ns: not significant; **** *p* < 0.0001 (determined using one-way ANOVA analysis). (**C**) The growth of the *C. neoformans* strains under different stress conditions. Yeast cells grown overnight were diluted to an OD_600_ value of 2.0, followed by ten-fold dilutions. 5-µL of each diluent was plated on YPD agar plates with different stressors and incubated at 30 °C for 2 days. The *C. neoformans* strains are indicated on the left, and the incubation conditions are on the top.

To confirm the role of Ade16 in *C. neoformans* virulence, we first examined the virulence factor production of i*ADE16 and ADE16*^OE^ strains in vitro. Interestingly, RNA interference of the *ADE16* gene led to a significant decrease in capsule size, while overexpression led to a significant increase in capsule production in *C. neoformans* (Figure 4A,B). The average relative size of the capsule produced by the i*ADE16* strain or the *ADE16*^OE^ strain was reduced by 35% or increased by 111%, respectively, compared with the H99 strain or *fbp1*Δ mutants (Figure 4B). This decrease or increase in capsule production is statistically significant (*p* < 0.0001) based on a one-way ANOVA analysis, indicating that Ade16 plays an important role in *C. neoformans* capsule production. We also examined the melanin formation of each *C. neoformans* strain and found that neither *ADE16* interference nor overexpression affected melanin formation in *C. neoformans* (Figure S2). 

Meanwhile, we also investigated the growth of the i*ADE16* strain and the *ADE16*^OE^ strain under different stress conditions. The results showed that the i*ADE16* strain was sensitive to 1.5 M NaCl but not sensitive to cell integrity-targeting chemicals, such as SDS and Congo red, suggesting that Ade16 may play a role in maintaining the osmotic pressure in *C. neoformans* cells (Figure 4C). 

### 3.6. Ade16 Regulates Sexual Reproduction of C. neoformans

**Figure 5:**
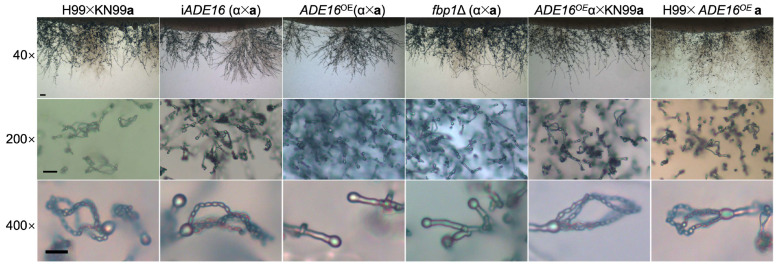
Ade16 is involved in the sexual reproduction of *C. neoformans*. The wild types, i*ADE16* interference strains, and *ADE16*^OE^ strains were crossed on MS medium for bilateral or unilateral mating assays. Mating hyphae and basidiospores at ×40 magnification (top panel, bar = 100 μm), ×200 magnification (middle panel, bar = 50 μm), and ×400 magnification (bottom panel, bar = 10 μm) were imaged after 2 weeks of incubation at 25 °C in the dark.

Our previous study showed that knockout of the *FBP1* gene blocked the basidiospore production in bilateral mating between *fbp1*Δ mutants. Since Ade16 is a downstream substrate of Fbp1, we speculated that Fbp1 might affect *Cryptococcus* sexual reproduction by regulating Ade16. To investigate the role of Ade16 in fungal mating, we constructed the i*ADE16* interference strains and *ADE16*^OE^ overexpression strains in both H99 and KN99**a** strain backgrounds. The development of dikaryotic hyphae and basidiospores was examined in bilateral mating in i*ADE16* interference strains or *ADE16*^OE^ overexpression strains. As with the wild-type strains, the i*ADE16* interference strains generated normal mating hyphae and morphologically normal-looking basidiospores (Figure 5). However, the bilateral mating between *ADE16*^OE^ overexpression strains generated normal mating hyphae but failed to produce spores, consisting with the phenotype of the *fbp1*Δ mutants (Figure 5), suggesting Fbp1 may regulate the sexual reproduction by regulating the Ade16.

**Figure 6:**
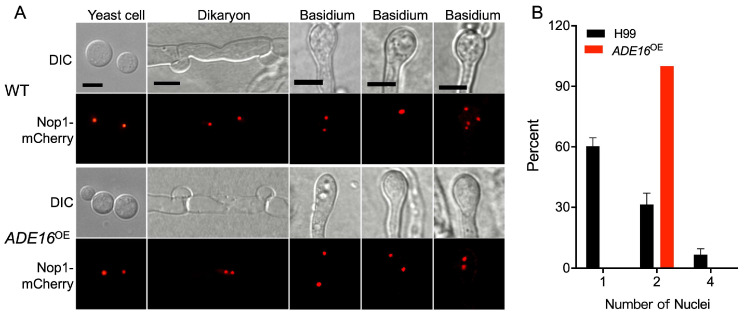
Fungal nuclei development in *ADE16*^OE^ overexpression strains. (**A**) Yeast cells, dikaryons, and basidia of the wild types or *ADE16*^OE^ overexpression strains were isolated and observed by a confocal microscope after incubation on MS medium for 1 or 2 weeks in the dark at 25 °C. DIC, differential interference contrast; WT, wild-type strains H99 and KN99**a**. Bars = 5 µm. (**B**) Statistical results of nuclei in basidium of wild types or *ADE16*^OE^ overexpression strains.

To explore the mechanism of the failure of *ADE16*^OE^ overexpression strains to produce basidiospores, we constructed a nuclear-located Nop1-mCherry fusion expression vector. We introduced it into both α and **a** mating types of the wild type and *ADE16*^OE^ overexpression strains to monitor the fungal nuclei positioning at different stages of mating in *C. neoformans*. The wild types (TBL101 and TBL102) or the *ADE16*^OE^ overexpression strains (TBL445 and TBL446) expressing the Nop1-mCherry were crossed, respectively, and their nuclear positioning was monitored during the mating. As shown in Figure 6A, a single nucleus was visualized in the yeast cells of both the wild types and the *ADE16*^OE^ overexpression strains, while two separated nuclei were found in each dikaryon produced after cell fusion. During mating, the wild-type strains had a single fused nucleus in the young basidium and four nuclei in each mature basidium, consistent with our previous findings [16]. However, the nuclei of the *ADE16*^OE^ overexpression strains failed to fuse in the bilateral mating, and only two separated nuclei were observed in both the young and the mature basidia (Figure 6A,B). These findings suggested that Ade16 influences meiosis during mating, which may help explain why the *ADE16*^OE^ overexpression strains could not produce basidiospores when crossed with a partner in bilateral mating. However, when grown in rich media, the growth rate and nuclear division of the *ADE16*^OE^ overexpression strains were normal, suggesting that Ade16 is not involved in the cell cycle during mitosis.

Taken together, we identified a downstream substrate of Fbp1, the AICAR transformylase/IMP cyclohydrolase Ade16, and found that Fbp1 regulates the sexual reproduction of *C. neoformans* by regulating ubiquitination degradation of its downstream substrate, Ade16.

## 4. Discussion

The F-box proteins are a critical component of the SCF E3 ligases and play a vital role in fungal virulence and development. Our previous studies have shown that the F-box protein Fbp1 is required for the sexual reproduction of the human fungal pathogen *C. neoformans*, as basidiospore formation is blocked in bilateral mating of *fbp1*Δ mutants [19]. However, the mechanism by which Fbp1 regulates sexual reproduction in *C. neoformans* remains unclear. To identify the potential downstream targets in Fbp1, we carried out an iTRAQ analysis coupled with LC-MS/MS in a previous study to investigate the enriched target proteins in *fbp1*Δ mutants [24]. In this study, we identified the AICAR transformylase/IMP cyclohydrolase Ade16 and demonstrated that Ade16 interacts directly with Fbp1, and that the degradation and ubiquitination of Ade16 depend on the function of Fbp1. The gene expression analysis revealed that the *ADE16* gene was expressed at all developmental stages, and the Ade16 protein was localized in the cytoplasm of *C. neoformans* cells. RNA interference of *ADE16* led to a significant decrease in capsule size, while overexpression led to a significant increase in capsule size (confirming Wizrah and colleagues’ results [22]) in *C. neoformans*. Overexpression of *ADE16* resulted in the production of normal mating hyphae but a failure of basidiospore formation, which is consistent with the phenotype of the *fbp1*Δ mutant (Figure 6), suggesting Fbp1 may regulate sexual reproduction by regulating Ade16. Our findings suggest that the SCF(Fbp1) E3 ligase-mediated UPS pathway regulates *C. neoformans* sexual reproduction by regulating Ade16.

We first analyzed the *ADE16* gene expression and found that the *ADE16* was expressed at all development stages of *C. neoformans*, and the Ade16 protein was localized in the cytoplasm of *C. neoformans* cells, consisting with the Ade17p localization in *S. cerevisiae* [32]. Since Ade16 was highly enriched in the *fbp1*Δ mutant and could be a downstream target of Fbp1, we then performed protein interaction, stability, and ubiquitination assays. We found that Ade16 interacts with Fbp1, and its stability and ubiquitination is dependent on the function of Fbp1. These findings suggest that the SCF(Fbp1) E3 ligase-mediated UPS pathway might regulate the purine de novo biosynthesis pathway in *C. neoformans* by regulating the ATIC Ade16. So far, to our knowledge, there have been no reports on the regulation of Ade16 protein degradation, so our findings may establish the regulatory pathway for Ade16 protein degradation.

The polysaccharide capsule is a virulence factor that plays a key role in the interaction between *C. neoformans* cells and the immune system, protecting *C. neoformans* cells from being phagocytosed by immune cells [33].In this study, we found that loss of *ADE16* expression decreased the capsule size of *C. neoformans*, while *ADE16* overexpression enlarged the capsule, indicating that formation of *C. neoformans* capsule is related to the expression level of *ADE16*. In the study by Wizrah et al., the *ade16*Δ strain also displayed reduced capsule size at 30 °C and 37 °C [22]. The results from both groups suggested that Ade16 plays an important role in the capsule formation of *C. neoformans*. 

Purines are ubiquitous life-sustaining biomolecules and can be generated by the de novo purine biosynthesis pathway [34,35]. Inosine monophosphate (IMP) is the common intermediate production in purine biosynthesis and can be converted into the purine nucleotides AMP or GMP using additional GTP or ATP [35]. Therefore, as a core enzyme in the de novo purine biosynthesis pathway, changes in the abundance of Ade16 affect the production of IMP, which in turn affects the formation of AMP and, thus, ATP. Nucleotide sugars are essential for polysaccharide synthesis [36], which requires a large amount of energy, meaning that any AMP or ATP depletion will negatively affect polysaccharide formation. Additionally, cAMP, synthesized from ATP through a cyclization reaction catalyzed by adenylate cyclase, is an important intracellular second messenger molecule regulated in many physiological processes [37]. In *C. neoformans*, studies reveal that the Gpa1-cAMP pathway regulates capsule induction in response to environmental stimuli [38]. Thus, interference of *ADE16* leads to a decrease in AMP, resulting in a significant decrease in capsule size, while overexpression leads to a significant increase in AMP and capsule size in *C. neoformans*. In Wizrah et al.’s study, due to adenine supplementation, the capsule defect observed in the *ade16*Δ mutant is much milder than the one we observed.

Sexual reproduction allows genetic material from two parents to recombine, resulting in recombinant offspring with the potential for variable adaptation, and allows natural selection to more effectively remove harmful mutations that accumulate in the parental genomes [39]. In *C. neoformans*, sexual reproduction links to virulence, antifungal drug resistance, and rapid adaptive evolution [40,41]. Many environmental factors and genetic circuits regulate sexual reproduction in *C. neoformans* [10]. Our previous study showed that the SCF(Fbp1) E3 ligase-mediated UPS regulates sexual reproduction in *C. neoformans* [19]. However, the molecular mechanism by which Fbp1 regulates sexual reproduction in *C. neoformans* remains unclear. In this study, we identified the AICAR transformylase/IMP cyclohydrolase (ATIC) Ade16 as a substrate of Fbp1 and demonstrated that Ade16 is involved in the sexual reproduction of *C. neoformans*. Ade16 is involved in the sexual reproductive process of *C. neoformans*, which is truly interesting. How Ade16 is involved in the sexual reproductive process of *C. neoformans* is unknown. There are few studies on Ade16 in fungi, mainly in *S. cerevisiae* [32,42,43,44]. In *C. neoformans*, Ade16 was identified by Wizrah et al. and proved to be essential for de novo purine biosynthesis [22]. The *ADE16* gene was only mentioned in research on a new high-throughput screening procedure for the detection of sporulation defects in 624 non-lethal homozygous deletion mutants created by the European Joint research project EUROFAN [45]. So far, the mechanism of Ade16’s involvement in the regulation of fungal sexual reproduction remains unclear and will be the subject of future studies.

In conclusion, our study discovered a novel substrate of Fbp1 in *C. neoformans* and unveiled a novel sexual reproduction regulatory pathway involving the SCF(Fbp1) E3 ligase-mediated UPS and its regulation of the AICAR transformylase/IMP cyclohydrolase Ade16 in *C. neoformans*. Given the importance of Ade16 in purine metabolism, it will be interesting to investigate how this gene regulates sexual reproduction in *C. neoformans*.

## Figures and Tables

**Table 1:** The partial enriched proteins identified in the *fbp1*Δ mutant of *C. neoformans*.

Accession	Description	Average *fbp1*Δ/H99	No. of PEST Domain
CNAG_05514	Uncharacterized protein	1.899038	0
CNAG_01974	Ribosomal protein	1.631707	2
CNAG_06195	Uncharacterized protein	1.595274	0
CNAG_00700	ATIC Ade16	1.544354	0
CNAG_05498	Uncharacterized protein	1.541769	1
CNAG_02860	Endo-1,3(4)-beta-glucanase	1.522101	2
CNAG_01019	Superoxide dismutase	1.470508	0
CNAG_02344	Uracil phosphoribosyltransferase	1.431079	1
CNAG_02738	Uncharacterized protein	1.400934	0
CNAG_01109	Uncharacterized protein	1.384226	1

## Data Availability

All relevant data are within the manuscript.

## Supplementary Materials

The following supporting information can be downloaded at: https://www.mdpi.com/article/10.3390/jof9070699/s1, Figure S1: Detection of the GFP-Ade16 fusion proteins; Figure S2: Ade16 is not involved in melanin production of *C. neoformans*. Table S1: Strains and plasmids used in this study; Table S2: Primers used in this study.
