# Supplementary material for: Ubiquitin Degradation of the AICAR Transformylase/IMP Cyclohydrolase Ade16 Regulates the Sexual Reproduction of Cryptococcus neoformans
Source: J Fungi (Basel). 2023 Jun 24;9(7):699. doi: 10.3390/jof9070699 (PMC10381356; doi:10.3390/jof9070699)
Supplement: Supplementary file 1 [file jof-09-00699-s001.zip › jof-2405103-supplementary.pdf]

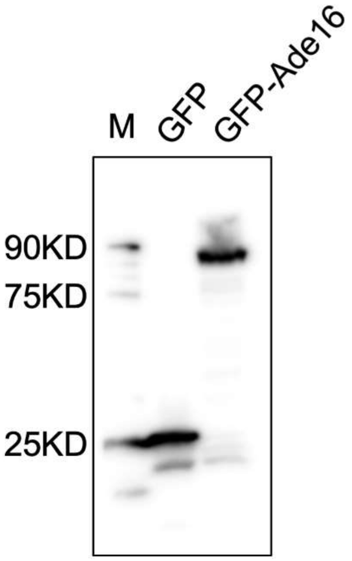

Figure S1. Detection of the GFP-Ade16 fusion proteins.

The expression of the GFP-Ade16 fusion protein in TBL308 was confirmed by Western blotting. M: protein marker.

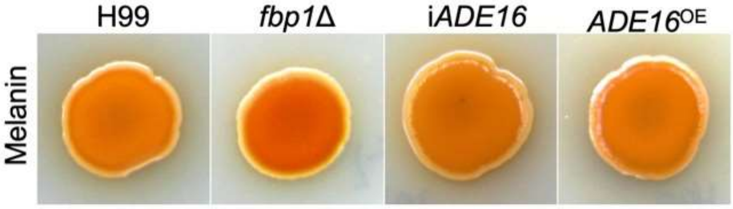

Figure S2. Ade16 is not involved in melanin production of *C. neoformans*.

Melanin production by the *C. neoformans ADE16* interference or overexpression strains was induced on Niger seed plates and photographed after incubation at 37°C for two days.

Table S1. Strains and plasmids used in this study

Strains/plasmids	Genotype/properties	Source/reference
E. coli		
DH5α	cloning strain	
C. neoformans		
H99	*MAT*α	Perfect et al., 1993
KN99**a**	*MAT***a**	Nielsen et al., 2003
TBL3	*MAT*α *fbp1*Δ::*NEO*	In this study
TBL81	*MAT*α Fbp1-Flag::*URA5*	In this study
TBL248	*MAT*α Fbp1-Flag::*URA5* P_{CTR4}-Ade16-HA::*NAT*	In this study
TBL264	*MAT*α P_{CTR4}-Ade16-HA::*NAT*	In this study
TBL265	*MAT*α *fbp1*Δ::*NEO* P_{CTR4}-Ade16-HA::*NAT*	In this study
TBL270	*MAT*α P_{CTR4}-Ade16::*NAT*	In this study
TBL288	*MAT*α P_{ACTIN}-Ade16-HA::NAT	In this study

TBL302	*MAT***a** P_{ACTIN}-Ade16-HA::NAT	In this study
TBL308	*MAT*α P_{H3}-GFP-Ade16::NAT	In this study
TBL309	*MAT***a** P_{H3}-GFP-Ade16::NAT	In this study
TBL310	*MAT*α P_{ADE16}-mCherry::NAT	In this study
TBL378	*MAT***a** P_{ADE16}-mCherry::NAT	In this study
TBL414	*MAT*α i*ADE16*::*NAT*	In this study
TBL415	*MAT***a** i*ADE16*::*NAT*	In this study
TBL445	*MAT*α *ADE16*^{OE}::*NAT* Nop1-mCherry::*NEO*	In this study
TBL446	*MAT***a** *ADE16*^{OE}:: NAT Nop1-mCherry::*NEO*	In this study
S. serevisiae		
YS1	NMY32	In this study
YS2	NMY32::AD-T7 BD-53	In this study
YS3	NMY32:: AD-T7 BD-LAM	In this study
YS5	NMY32:: AD-Fbp1 BD-Ade16	In this study
YS9	NMY32:: AD BD-Fbp1	In this study
YS16	NMY32:: AD BD-Ade16	In this study
YS17	NMY32:: AD-Ade16 BD-Fbp1	In this study
Plasmids		
pCN19	Amp^r Plasmid harboring *GFP* under histone H3 promoter	Price et al., 2008
pTBL5	Amp^r Plasmid harboring mCherry-GPD1 terminator and *NAT* marker	In this study
pTBL59	Amp^r Vector for P_{ACTIN}-Nop1-mCherry-NEO for nuclear positioning	In this study
pTBL100	$Kana^r$ Vector for pGBKT7-FBP1 for yeast two-hybrid	In this study
pTBL106	Amp^r Vector for pGADT7-ADE16 for yeast two-hybrid	In this study
pTBL142	Amp^r Vector for pGADT7-FBP1 for yeast two-hybrid	In this study
pTBL145	$Kana^r$ Vector for pGBKT7-ADE16 for yeast two-hybrid	In this study
pTBL149	Amp^r Vector for P_{CTR4}-ADE16-HA-NAT for Ade16 stability assay	In this study
pTBL153	Amp^r Vector for P_{ACTIN}-Grp1-HA-NAT for *GRP1* overexpression	In this study
pTBL174	Amp^r Vector for P_{ACTIN}-Ade16-HA-NAT for Ade16 overexpression	In this study
pTBL186	Amp^r Vector for P_{H3}-*GFP*-Ade16-NAT for Ade16 localization	In this study
pTBL196	Amp^r Vector for P_{ADE16}-mCherry-NAT for temporal expression assay	In this study
pTBL237	Amp^r Vector for *ADE16* RNAi	In this study

Table S2. PCR primers used in this study

Primers	Description	Sequence (5'-3')
TL17	M13 F	GTAAAACGACGGCCAG
TL18	M13 R	CAGGAAACAGCTATGAC
TL19	*NEO* split F	GGGCGCCCGGTTCTTTTTGTCA
TL20	*NEO* split R	TTGGTGGTCGAATGGGCAGGTAGC
TL59	*NEO* R4	TGTGGATGCTGGCGGAGGATA
TL67	*STE20A* α F	CCAAAAGCTGATGCTGTGGA
TL68	*STE20A* α R	AGGACATCTATAGCAGAT
TL69	*STE20A* a F	TCCACTGGCAACCCTGCGAG
TL70	*STE20A* a R	ATCAGAGACAGAGGAGCAAGAC
TL367	*ADE16* KO F1	ACATGACGAGCACAGCCAGTAGCA
TL368	*ADE16* KO R1	CTGGCCGTCGTTTTACGGTCGCCCCCGTCTCCAC
TL369	*ADE16* KO F2	TGCGAAGTGCTGGATAGATGAAGATG
TL370	*ADE16* KO R2	AGACCCTTCACCCCGCTGTTCACG
TL371	*ADE16* KO F3	AGACCCTTCACCCCGCTGTTCACG

TL372	*ADE16* KO R3	TCCACTGGGGGCTGCGAGGTAGGT
TL373	*ADE16* KO F4	ATCTCGCTGCACTCTTCTCAAATA
TL572	Fbp1-BD F	CCGAATTCCCGGGGATCCACATGCCCGTACGACCGTCAAGAAGC
TL573	Fbp1-BD R	GTTATGCGGCCGCTGCAGTCAACGTCCGTTACCGAATCGTTG
TL588	Ade16-AD F	GGCATCGATACGGGATCCACATGTCTTCCGAGGCTCCTATCGGT
TL589	Ade16-AD R	ATTCATCTGCAGCTCGAGTTAATGGTGGAACTGGAGATGATT
TL879	Fbp1-AD F	GGCATCGATACGGGATCCACATGCCCGTACGACCGTCAAGAAGC
TL880	Fbp1-AD R	ATTCATCTGCAGCTCGAGTCAACGTCCGTTACCGAATCGTTG
TL883	Ade16-BD F	CCGAATTCCCGGGGATCCACATGTCTTCCGAGGCTCCTATCGGT
TL884	Ade16-BD R	GTTATGCGGCCGCTGCAGTTAATGGTGGAACTGGAGATGATT
TL893	P_{CTR4}-Ade16-HA F1	TCCTGCAGCCCGGGGGATCCATGTCTTCCGAGGCTCCTATCGGT
TL894	P_{CTR4}-Ade16-HA R1	ACGTCGTATGGGTAGGATCCATGGTGGAACTGGAGATGATTG
TL1036	*ADE16* P_{CTR4} REP F1	TATCGGGCAGCAGAGGGCAGAG
TL1037	*ADE16* P_{CTR4} REP R1	CTGGCCGTCGTTTTACGTGAGTGAACCACCACAGTCAGAT
TL1034	*ADE16* P_{CTR4} REP F2	GTCATAGCTGTTTCCTGGCCAAATCAAGCTTATCGATGGTC
TL1038	ADE16 P_{CTR4} REP R2	TCGTCGAGGGGAAGACCGACGG
TL1092	P_{ACTIN}-Ade16-HA F1	CGCCCAACATGTCTGGATCCATGTCTTCCGAGGCTCCTATCGGT
TL1093	P_{ACTIN}-Ade16-HA R1	ACGTCGTATGGGTAGGATCCATGGTGGAACTGGAGATGATTG
TL1164	GFP-Ade16 F	GACGAGCTGTACGGATCCATGTCTTCCGAGGCTCCTATCGGT
TL1165	GFP-Ade16 R	CTGGCGGCCGTTACTAGTTTAATGGTGGAACTGGAGATGATT
TL1166	P_{ADE16}-mCherry F	ACGGTATCGATAAGCTTCTGTTCGTGATGGCGAGAGAACAT
TL1167	P_{ADE16}-mCherry R	CTAGAACTAGTGGATCCGTTGCTTGTATGTGGATGAGGTTG
TL1248	*ADE16* qRT-PCR F	CAAGAAAGGCACCAAGCG
TL1249	ADE16 qRT-PCR R	GGGGAAGGGGAAAAAGG
TL1334	*ADE16* Intro F	AACTCGCCCAACATGTCTGGATCCGTGAGTTGTTTCTCCCGC
TL1335	*ADE16* Intro R	CTACTGCTACTGTAACCCTTAATTAAACTAGTCTGTTTGATGTCAAGAGG
TL1336	*ADE16* (RNAi) 5'-3' F	AACTCGCCCAACATGTCTGGATCCGGATGTTTCCAACATCACCA
TL1337	*ADE16* (RNAi) 5'-3' R	GCGGGAGAAACAACTCACGGATCCGAAGTAATCACTGATAGCCTCG
TL1340	*ADE16* (RNAi) R	GGATAACGGAGAAAGAGGAAATCA
TL1467	*ADE16* RNAi reverse F1	CCTTAATTAAGGATGTTTCCAACATCACCA
TL1468	*ADE16* RNAi reverse R1	GGACTAGTTTAGAAGTAATCACTGATAGCCTCG
